# Novel Therapeutic Strategies in Alzheimer’s Disease: Pitfalls and Challenges of Anti-Amyloid Therapies and Beyond

**DOI:** 10.3390/jcm13113098

**Published:** 2024-05-25

**Authors:** Giacomo Tondo, Fabiola De Marchi, Francesca Bonardi, Federico Menegon, Gaia Verrini, Davide Aprile, Matteo Anselmi, Letizia Mazzini, Cristoforo Comi

**Affiliations:** 1 Neurology Unit, Department of Translational Medicine, Maggiore della Carità Hospital, University of Piemonte Orientale, 28100 Novara, Italy; giacomo.tondo85@gmail.com (G.T.); 20051713@studenti.uniupo.it (F.B.); 20042535@studenti.uniupo.it (F.M.); 20052587@studenti.uniupo.it (G.V.); aprile.davide@hotmail.com (D.A.); 20029549@studenti.uniupo.it (M.A.); letizia.mazzini@uniupo.it (L.M.); 2 Neurology Unit, Department of Translational Medicine, Sant’Andrea Hospital, University of Piemonte Orientale, Corso Abbiate 21, 13100 Vercelli, Italy; cristoforo.comi@med.uniupo.it; 3 Interdisciplinary Research Center of Autoimmune Diseases (IRCAD), University of Piemonte Orientale, 28100 Novara, Italy

**Keywords:** cognitive impairment, dementia, neuroinflammation, therapy, amyloid, tau, aducanumab

## Abstract

Alzheimer’s disease (AD) causes a significant challenge to global healthcare systems, with limited effective treatments available. This review examines the landscape of novel therapeutic strategies for AD, focusing on the shortcomings of traditional therapies against amyloid-beta (Aβ) and exploring emerging alternatives. Despite decades of research emphasizing the role of Aβ accumulation in AD pathogenesis, clinical trials targeting Aβ have obtained disappointing results, highlighting the complexity of AD pathophysiology and the need for investigating other therapeutic approaches. In this manuscript, we first discuss the challenges associated with anti-Aβ therapies, including limited efficacy and potential adverse effects, underscoring the necessity of exploring alternative mechanisms and targets. Thereafter, we review promising non-Aβ-based strategies, such as tau-targeted therapies, neuroinflammation modulation, and gene and stem cell therapy. These approaches offer new avenues for AD treatment by addressing additional pathological hallmarks and downstream effects beyond Aβ deposition.

## 1. Introduction

### 1.1. Pathogenic Insight into the Spectrum of Neurodegenerative Diseases

Neurodegenerative diseases (NDDs) are neurological disorders characterized by selective and progressive synaptic dysfunction and neuronal loss. The central nervous system (CNS) is a highly complex structure where the cells fail to regenerate after damage. In NDDs, the topographical distribution of damage and the specific neuronal population affected by degeneration reflect different pathologies and symptoms. Pathogenesis in these disorders is often linked to abnormal protein accumulation, which may contribute to progressive neuronal dysfunction and death through several mechanisms including oxidative stress, excitotoxicity, mitochondrial dysfunctions, DNA damage, and neuroinflammation [1,2].

Based on the pathological protein deposition, the concept of proteinopathies has been introduced, describing a group of NDDs sharing the same misfolded protein accumulation process and aiding the development of precisely targeted therapeutic strategies. The identified protein-associated spectra are the Alzheimer’s disease (AD) spectrum, tauopathies, the α-synuclein spectrum, and prion-related encephalopathies [3]. Identifying specific biomarkers in each pathological condition supports the diagnostic process and the prognostic stratification of patients, opening novel therapeutic perspectives [4]. However, to date, no therapeutic agents can promote neuronal regeneration in damaged brain regions. 

A further common pathogenic mechanism among NDDs is neuroinflammation. Acute neuroinflammation is physiological in the immediate repair of brain tissues exposed to environmental insults. Conversely, a persistent inflammatory response is typically associated with the development and progression of several brain disorders, including NDDs [5]. A well-known central mechanism of neuroinflammation linked to NDDs is related to microglial activation, with the production of pro-inflammatory cytokines such as interleukin (IL) 1 (IL-1), IL-6, IL-18, tumor necrosis factor (TNF), nitric oxide (NO), and reactive oxygen species (ROS) [6,7]. Microglia activation can be secondary to different agents, including infections, foreign pathogens, lipopolysaccharide, prions, or, in NDDs, pathologically modified CNS proteins such as β-amyloid (Aβ), tau, and α-synuclein [8,9,10]. A high level of cytokines causes an increase in blood–brain barrier (BBB) permeability associated with the expression of adhesion molecules and consequent recruitment of circulating myeloid blood cells (monocytes, macrophages, and dendritic cells) and T and B lymphocytes, which contribute to amplifying the inflammatory response [11]. The role of neuroinflammation in the pathogenesis of NDDs is also supported by several in vivo studies, showing the presence of microglia activation in the CNS by the use of positron emission tomography (PET) imaging of neuroinflammation [12,13,14] and altered neuroinflammatory markers in cerebrospinal fluid (CSF) and blood [15,16,17]. 

The link between neuroinflammation and neurodegeneration is a chain mechanism: neuroinflammatory reactions lead to neurodegeneration, and consequently, a neurodegenerative phenomenon induces further central inflammation [18]. Several factors can trigger neuroinflammatory responses in the CNS, including the normal aging process. During aging, microglia cells change morphology, increase inflammatory marker expression, and reduce neuroprotective agent expression. Consequently, disturbance of brain homeostasis, either directly via the deposition of abnormal proteins or cerebral hypo-perfusion or indirectly via peripheral inflammation, will activate microglia to synthesize several pro-inflammatory agents, which may lead to inflammation and cell death [19]. 

Another critical topic is inflammasome. Inflammasomes are a group of multimeric signaling complexes that include a sensor Nod-like receptor (NLR) molecule, the adaptor protein ASC, and caspase-1. The NLRP3 inflammasome is currently the best-characterized inflammasome. Multiple signals, which are potentially provided in combination and include endogenous danger signals and pathogens, trigger the formation of an active inflammasome, which, in turn, will stimulate the cleavage and the release of bioactive cytokines, including IL-1β and IL-18. These responses start inflammatory signaling cascades, contributing to neuronal injury, cell death, and neuroinflammation [20]. 

### 1.2. The Paths to Alzheimer’s Disease Therapy 

AD is the most common NDD and the most common cause of neurodegenerative dementia, accounting for about 60–80% of cases. It is estimated that 6.9 million Americans over 65 years are living with AD in 2024, and, across the world, more than 55 million people are suffering from dementia (https://www.alz.org/media/documents/alzheimers-facts-and-figures.pdf; accessed on 13 April 2024). AD dementia is typically characterized by an insidious onset of memory disturbances, generally affecting people aged more than 65 years in so-called late-onset AD. AD dementia can also manifest in people aged less than 65 years, in the early-onset subtype, which consists of 5% of AD cases and differs from the usual amnestic presentation of typical AD, involving other cognitive domains than memory, such as the visuospatial, executive, or language domain [21]. Disease duration usually lasts from four to eight years after the diagnosis. However, some people live for 20 years with AD [22,23,24]. Regardless of the onset, the progressive worsening of the cognitive deficit causes an impairment in other cognitive domains since the affected person is unable to perform autonomously the activities of daily living, needing caregiving. The development of a severe cognitive impairment often causes complications, including bed rest, swallowing deficit, malnutrition, and infections such as pneumonia, which usually lead to death [25].

From a biological point of view, the pathological hallmarks of AD are the extracellular senile plaques, mainly composed of Aβ fragment deposition, and the intracellular neurofibrillary tangles, primarily composed of hyperphosphorylated-tau (p-tau) [26]. The “amyloid cascade” hypothesis identifies Aβ as the leading player in triggering the neurodegenerative changes in AD [27]. The Aβ peptides derive from the cleavage of the Amyloid Precursor Protein (APP) by different enzymes called secretases. The amyloidogenic pathway causes the overproduction of toxic Aβ peptides that form oligomers and fibrils and easily precipitate in the senile plaques [28]. In brief, the APP is cleaved by α- and β-secretase to originate fragments of 83 (C83) or 99 (C99) residues, respectively. Then, the APP-C99 is cleaved by γ-secretase into 48-residue (Aβ-48) or 49-residue (Aβ-49) peptide. The γ-secretase further acts on the Aβ-48 and Aβ-49, generating several peptides, including Aβ-40, Aβ-42, and Aβ-43, which are prone to aggregation into the amyloid plaques. In the non-amyloidogenic pathway, C83 is cleaved by the γ-secretase into p3 and the APP intracellular domain [29]. The importance of the amyloidogenic pathway has been demonstrated in individuals with familial AD, where, in most cases, APP mutations increase Aβ—and especially Aβ-42—production [30]. In addition, individuals affected by Down’s syndrome have three copies of the APP gene, which is located on chromosome 21. This aspect explains why Down patients almost invariably develop AD after 40 years of age [31]. 

In physio-pathological conditions, Aβ can be present in several intermediate aggregation forms, including oligomers, protofibrils, fibrils, and plaques [32]. Oligomers are involved in multiple physiological functions, including modulating intracellular signaling and synaptic activity [33]. Aβ-42 is less soluble than other peptides and more inclined to generate aggregates, which is an obstacle to the optimal function of Aβ proteins [34]. Also, the intermediate soluble oligomers share a toxic potential. It has been reported in animal models that the injection in the mouse hippocampus of Aβ oligomers is associated with synaptic alterations, including dendritic spine abnormalities and impaired learning [35]. This noxious potential is shared by both low-number and high-number Aβ oligomers [32]. The protofibrils are large soluble Aβ aggregates able to negatively modulate synaptic plasticity in the mouse hippocampus, affecting learning [36]. The protofibrils primarily act on microglia activity, contributing to generate a deleterious neuroinflammatory response [37]. Protofibrils are prone to generating Aβ fibrils with the characteristic β-sheet structure, which is associated with different solubility levels and toxicity. Fibrils and plaques, the last steps of Aβ aggregation, have been associated with neurite disruption, decreased spine density, synaptic dysfunction, and neuronal loss [38,39]. In addition, the presence of Aβ forms, including oligomers, protofibrils, and fibrils, stimulates neuroinflammation and microglia activation, which can contribute to neurodegenerative changes [40].

Based on these findings, the toxic proteinopathy hypothesis suggests that Aβ is involved in a gain-of-function mechanism, and, since Aβ deposition is associated with AD degenerative changes, lowering Aβ levels would counteract neurodegeneration and cognitive decline [41]. However, despite decades of research, the disappointing results of recent clinical trials based on strategies aiming at counteracting Aβ deposition or favoring Aβ clearance stimulate a critical evaluation of the amyloid cascade hypothesis [42]. The main criticism in identifying the Aβ pathway as the trigger of neurodegeneration is linked to the evidence that Aβ deposits scarcely correlate with cognition, that Aβ deposits can also be detected in cognitively unimpaired individuals [43,44], and that markers of neuronal injury and tau pathology can be independent of Aβ deposition [45]. In contrast to the gain-of-function mechanism, the hypothesis of a protein loss-of-function has been developed, also supported by translational and genetic studies [41]. The Aβ formation of brain aggregates implies another mechanism beyond protein accumulation, that is protein depletion in fluid. The depletion of Aβ soluble forms can also be advocated as a possible crucial mechanism in neurodegeneration, since several studies have reported that Aβ-42 low CSF levels are associated with the longitudinal development of AD symptoms and with neurodegenerative markers, and that low Aβ levels better correlate with cognitive decline than the burden of the insoluble form [46,47,48]. The loss-of-function hypothesis is supported also by the evidence that high levels of soluble Aβ-42 in individuals with brain abnormal amyloid load are associated with normal cognition, in both sporadic and genetic forms of AD [49,50]. The gain-of-function and the loss-of-function hypothesis, supporting the protheinopaty or the proteinopenia mechanism, respectively, are not necessarily exclusive, since the Aβ deposition is associated with Aβ soluble form depletion and vice versa, and AD pathogenesis is likely to involve multiple molecular pathways whether or not associated with amyloid.

The complexity of the involved pathways and the incomplete clarification of the amyloid cascade and its consequences partly explain the disappointing results of the anti-amyloid therapeutic approaches. There is strong evidence that the oligomers are the main toxic Aβ species in AD [51]. The severity of neurodegenerative changes does not correlate with the load of senile plaques but with soluble Aβ, and oligomers are cytotoxic and damage synapsis in vitro [52]. Thus, targeting oligomers, protofibrils, fibrils, and plaques implies additional variability in the therapeutic response. In addition, the “amyloid cascade” begins decades before the symptom’s onset, and it represents only a part of the multiple molecular alterations characterizing AD, including tau-mediated toxic effect and neuroinflammation. As a consequence, the current anti-Aβ therapeutic approaches might be too little and too late to counteract AD [53]. The advances in pharmacological anti-Aβ approaches culminated with the recent development of anti-Aβ antibodies, including lecanemab, aducanumab, and solanezumab, showing the possibility of binding oligomers and monomers rather than fibrils and senile plaques. The identification of early molecular alterations, along with the validation of biomarker-based early diagnosis, would accelerate the process of disease-modifying drug development, allowing the targeting of altered molecular pathways before the occurrence of irreversible neurodegenerative changes. However, amyloid represents only one side of the multifaced pathogenic AD process.

In this vein, beyond the recognized role of Aβ in AD pathogenesis, evidence from the last twenty years supports the role of tau, whose deposition has been more strictly associated with the development of neuronal dysfunction and cognitive impairment [54]. While Aβ pathology may interfere with synaptic activity, tau pathology affects neuronal stability and survival, both acting with a synergic effect on neurodegeneration [55]. 

Tau is a microtubule-associated protein, existing in six molecular isoforms in the adult human brain, coded by a single gene on chromosome 17. Tau protein plays a role in stabilizing microtubules and regulating axonal transport. In physiological condition, tau phosphorylation regulates the binding to microtubules, while in AD, tau abnormal hyperphosphorylation favors its deposition into neurofibrillary tangles [56]. The topographical distribution of neurofibrillary tangles follows a precise spatiotemporal pattern in AD, initially involving medio-temporal regions and subsequently spreading to the neocortex and reflecting the progression of cognitive impairment [57]. In fact, the accumulation of abnormal tau causes neuronal loss by promoting mitochondrial damage, synaptic dysfunction, and microglia-mediated neuroinflammation [58]. The correspondence between tau deposition and clinical impairment makes anti-tau therapy a promising approach in AD. Nevertheless, targeting tau is challenging due to different tau isoforms and to the presence of numerous post-translational modifications [59], and, to date, it is not perfectly clear whether targeting the N- or the C-terminal would reduce tau toxicity [60]. 

In addition to pathological protein deposition, microglia activation, and deleterious neuroinflammatory responses have been proposed as potential triggers of neurodegeneration in AD [61]. In people carrying genetic mutations causing AD, Aβ and tau pathology starts to accumulate decades before the symptom’s onset [62,63]. Similarly, markers associated with neurodegenerative changes, including brain atrophy and reduced glucose metabolism, can be detected in asymptomatic or pre-symptomatic individuals along the AD continuum [62,64]. These aspects led researchers to investigate biomarker changes, meaning changes that can be measured, indicating the presence or the absence of AD-related changes [65]. Identifying reliable biomarkers plays a double role in AD and in NDDs, improving the diagnostic accuracy in the preclinical and prodroma stages and aiding the development of potential therapeutic strategies [66]. 

## 2. Overview of Novel Therapeutic Approaches for Slowing or Preventing AD

**Figure 1:**
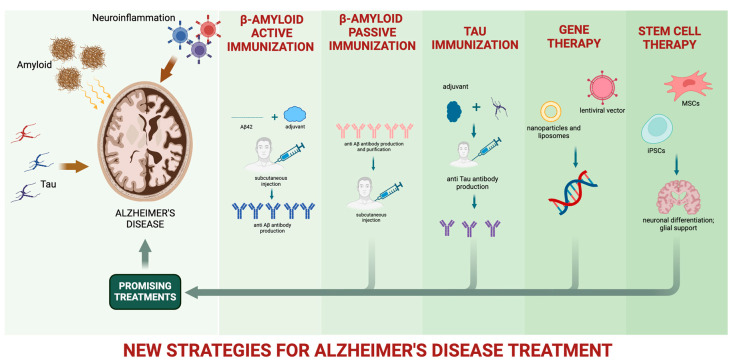
New strategies for AD pharmacological treatment.

Currently, available treatments for AD rely on drugs attenuating dementia progression and temporarily improving quality of life, providing only a modest symptomatic benefit to cognitive decline [67]. These drugs include the acetylcholinesterase inhibitors (donepezil, galantamine, and rivastigmine), aiming to enhance cholinergic neurotransmission, and memantine, an antagonist of N-methyl-D-aspartate receptors. In 2021, the anti-Aβ monoclonal antibody (mAb) aducanumab was approved by the Food and Drug Administration (FDA) for AD treatment, generating tremendous hope and sharp debate [68]. Numerous strides in pharmacological treatments for AD have yielded encouraging outcomes in recent years (Figure 1). Nevertheless, achieving satisfactory clinical benefits remains a distant goal [69].

Most molecules explored as potential targets for AD modifying treatment are involved in Aβ or p-tau production and in Aβ plaque and NFT formation. The Aβ cascade may be theoretically interrupted in different phases by inhibiting Aβ production, reducing Aβ aggregation, or favoring Aβ clearance [70]. In fact, enhancing Aβ clearance has been a largely explored approach [71], and the inhibition of Aβ generation may be obtained by regulating the activity of secretases involved in APP processing. Possible approaches—to date with unsatisfactory results—include activating the non-amyloidogenic pathway, stimulating the α-secretase, or inhibiting the amyloidogenic pathway by employing β-secretase 1 (β-site APP-cleaving enzyme, or BACE 1) or γ-secretase inhibitors [72,73].

Another possible approach is obstacle Aβ aggregation. Several small molecules have been studied for their anti-Aβ aggregation activity. Among natural molecules, polyphenols such as resveratrol and curcumin received particular attention for inhibiting Aβ fibril elongation. In addition, their antioxidant activity can be related to the possibility of reducing tau hyperphosphorylation and aggregation. Unfortunately, these molecules have low bioavailability, which reduces their clinical use [74].

An increased concentration of Aβ in the brain is associated with the development of synaptic dysfunction and neurodegenerative changes in all the phases of the AD continuum, from the preclinical to the overt-dementia phase [64,75,76,77]. Thus, despite several obstacles, active and passive immunization therapies, which are able to remove the Aβ product or to favor Aβ clearance, are the most studied approaches in AD. 

Active immunization is based on administering Aβ fragments that can trigger the natural immune response and produce antibodies against Aβ; it was effective in favoring Aβ clearance in mouse models [78]. However, the development of anti-Aβ vaccines implies some issues, including the following: the vaccines should break self-tolerance, considering that Aβ is also present in normal cells [79]; stimulating an autoimmune response may generate adverse effects, such as meningoencephalitis related to a T-cell-mediated reaction against Aβ [80]; the response to a vaccine may vary according to the individual-related factors, such as age and immune level [81,82], and according to vaccine-related factors, such as the used epitope (antibodies directed against one epitope may be more effective than others, for example, preferably favoring the elimination of brain Aβ plaques) [83,84]. Lastly, active immunization reactions can be difficult to modulate, control, or stop, especially in the elderly [85].

Passive immunotherapy employs humanized mAbs promoting Aβ clearance by several mechanisms, including the opsonization of the antigen, which causes macrophage phagocytosis and complement activation; the antibody-mediated modification of the Aβ monomer’s structure; blocking the formation of oligomers or fibrils; and the antibody-mediated peripheral reduction of Aβ, which promotes Aβ efflux from the central nervous system [86]. Clinical trials involving mAbs have been developed to promote Aβ clearance from the brain. MAbs target Aβ plaques against different epitopes of Aβ, but they are ineffective against monomers [87]. 

Similarly to Aβ, targeting tau is not extremely simple. Tau plays essential physiological functions; thus, the unconditioned blockage of its production may have significant side effects. In addition, tau exists in several isoforms, which makes identifying the right target to stop neurodegenerative changes challenging [73]. Selective inhibitors of tau-phosphorylation have been tested and shown to reduce tau aggregation together with Aβ deposition, but without clinical benefit [88]. Also, molecules that inhibit tau-fibrillation failed to induce clinical improvement [89].

Besides Aβ and tau pathology, neuroinflammatory responses have been linked to the development of neuronal dysfunction and cognitive impairment in AD [8,61,90]. Consequently, targeting neuroinflammatory responses may represent a decisive opportunity for counteracting AD development [91]. Early therapeutic approaches included the use of anti-inflammatory drugs [92], while the most recent include the following: (1) regulating the activity of specific inflammatory molecules and receptors, such as TNF-α [93], the triggering receptor expressed on myeloid cells 2 (TREM2) [94,95], and the family of receptor tyrosine kinases [96,97]; (2) modulating microglia responses to increase Aβ clearance and microglia phagocytic activity [98,99,100]; (3) enhancing astrocytes activity to protect BBB integrity and favor Aβ processing [101,102].

## 3. Amyloid-Beta Protein Immunotherapies

### 3.1. Anti-Amyloid Therapy

Both active and passive immunization allow the promotion of Aβ clearance. AN1792 was the first Aβ vaccine, based on a human full-length Aβ peptide associated with an adjuvant, reducing brain Aβ deposits and improving cognitive performance in mice [78]. The phase I trial based on AN1792 administration showed efficacy in reducing the amyloid load in humans. However, the phase II trial testing its efficacy and safety was stopped due to the high rate of meningoencephalitis (6% of treated patients) [80,103]. The patients involved in the study continued to be followed up to test the long-term effects of the vaccine on brain amyloid load and cognition [104]. Post-mortem evaluation of a few patients showed reduced amyloid pathology in treated individuals [105]. However, the progressive cognitive decline of patients despite amyloid clearance suggested that other processes should be involved in neuronal dysfunction [106,107]. 

Subsequent vaccines were developed with truncated Aβ fragments to elude T-cell activation and avoid meningoencephalitis. CAD106 consisted of Aβ1-6 fragments, and the phase I trial confirmed the efficacy of this strategy since no cases of meningoencephalitis after 52 weeks of treatment were reported [108]. UB311 has been developed linking the Aβ1-14 epitope to a delivery system stimulating T helper cell activation, which in turn favors a B cell response without stimulating the inflammatory response of T cells [109]. The phase II trial showed an excellent antibody response in AD patients with a good safety profile [110].

Most vaccines target the N-terminal of Aβ, while ABvac40 targets the C-terminal end of the Aβ40 peptide. In the phase I trial, ABvac42 showed a specific anti-Aβ40 antibody response in more than 90% of treated patients, with an excellent safety profile—no patients developed edema abnormalities or hemorrhages [111].

Active immunotherapy has a main advantage, which is stimulating the production of endogenous antibodies without repetitive administration. However, no significant clinical benefit has been reported in AD patients, and due to possible unpredictable immune response carrying on potentially severe side effects, no vaccine has yet been approved for marketing [107]. 

Passive immunotherapy employs humanized mAbs directly injected into the patient promoting Aβ clearance [71]. Studies in mouse models demonstrated that the repeated injections of an antibody directed against the N-terminal of Aβ can lower the brain Aβ levels [112]. Thus, several mAbs have been developed that can target different stages of Aβ plaque formation, favoring the clearance of Aβ plaques, such as aducanumab, bapineuzumab, and donanemab; targeting oligomers and protofibrils, such as lecanemab; or targeting monomers, oligomers, and fibrils, including ponezumab, crenezumab, and gantenerumab; lastly, solanezumab binds exclusively to Aβ monomers [113].

The use of mAbs is limited by the development of dose-dependent adverse effects, which can be visible in one-third of patients showing “amyloid-related imaging abnormalities” (ARIAs) [114]. ARIAs can be associated with the development of vasogenic edema (ARIA-E) or cerebral micro-hemorrhages (ARIA-H), characterized by the neuroimaging evidence of hemosiderin deposits. ARIAs were reported in clinical trials investigating the safety and efficacy of almost all mAbs, generally dose-dependent [115,116,117]. Currently, the FDA has approved the mAbs aducanumab and lecanemab for AD treatment, while donanemab is under evaluation for potential approval. 

Bapinezumab is a monoclonal antibody that preferentially binds Aβ fibrils and plaques [118]. It showed a good safety profile, but without clinical effectiveness [116]. Donanemab has a high affinity against Aβ plaques [119]. The only published data show no significant cognitive benefit [115], while a subsequent analysis reported a significant effect in slowing AD progression [113], keeping open the possibility for this molecule. Lecanemab preferentially binds oligomers and protofibrils [120]. The first clinical study with lecanemab reported that ascending doses of the drug (maximum 10 mg/kg biweekly for four months) were safe and well tolerated [121]. A subsequent phase II trial analysis [122] showed a reduced cognitive decline in the treated group compared with the placebo but without reaching the primary endpoint, despite significant benefit in removing brain Aβ plaques [122]. Ponezumab is a monoclonal antibody binding to the C-terminal of Aβ-40. In a phase III trial, no patients showed ARIA-E, while up to 25% of treated patients showed ARIA-H; in addition, no significant clinical effect was reported [123]. Crenezumab binds Aβ monomers, oligomers, and fibrils [124]. It has been shown to reduce brain amyloidosis by the evidence of reduced levels of Aβ oligomers in CSF [125]. However, the phase III trial was stopped due to unsatisfactory results [126]. Gantenerumab binds monomers, oligomers, and fibrils [120]. No clinical benefit has been reported in studies involving gantenerumab, with a high rate of ARIAs [127,128]. Solanezumab targets the mid-domain of soluble Aβ. The trial involving solanezumab was stopped due to unsatisfactory results, despite a very low rate of ARIAs (about 1–6%) [129,130,131]. In conclusion, in the field of passive AD anti-amyloid therapy, criticisms have been raised regarding the right target and the right disease stage, meaning that acting when neurodegenerative changes are already manifested could have potentially led to the trials’ failure. Clinical trials targeting Aβ are detailed in Table 1. 

### 3.2. The Case of Aducanumab

On 7 June 2021, aducanumab, an mAb targeting aggregated Aβ, was approved by the FDA for AD treatment. Transgenic animal model studies first provided evidence of aducanumab’s benefit in entering the brain, selectively binding aggregated Aβ forms while showing low affinity for soluble monomers and reducing Aβ load in a dose-dependent manner [132,133,134].

In humans, in the PRIME study, increasing intravenous doses of aducanumab were administered in patients with mild-to-moderate AD. Repeated amyloid PET scans showed marked brain amyloid load reduction after treatment, in a dose-dependent manner. In addition, aducanumab was effective in reducing cognitive decline as assessed by the Clinical Dementia Rating (CDR) Sum of Boxes and Mini Mental State Examination scores. The study also reported the dose-dependent side effect of ARIAs: ARIA-E occurred in a dose-dependent manner in a percentage between 3% (lowest aducanumab dose) and 41% (at the highest aducanumab dose, corresponding to 10 mg/kg) [132]. In most cases, ARIA-E led to no symptoms, and no patients were hospitalized. Nevertheless, about one-third of patients with ARIAs developed confusion, visual disturbances, or headache, and about a half of these cases dropped out of the trial [135].

Aducanumab was further tested in two identical trials, EMERGE and ENGAGE, recruiting over 3000 MCI and early AD patients [136]. In each study, aducanumab was administered at a low or high dose every four weeks. Originally designed to last 18 months, both trials were prematurely discontinued in 2019 due to a futility analysis predicting no effectiveness [137]. A subsequent analysis showed that the EMERGE high-dose treated group had met the primary endpoint, based on a significant reduction of cognitive decline as measured by the CDR Sum of Boxes. Conversely, the ENGAGE trial confirmed no significant effect of aducanumab on cognitive decline in the treated groups against a placebo. ARIA-E occurred in about one-third of individuals in the high-dose group overall, and more often in apoE4 carriers [136,138].

Following the re-evaluation of the EMERGE and ENGAGE trial results, in June 2021, the FDA approved aducanumab for medical use through the “Accelerated Approval Pathway” [139]. The accelerated approach was linked to the biological efficacy of aducanumab in reducing brain Aβ pathology and protecting against tau deposition, meeting the criteria for being a surrogate endpoint [140,141]. Initially approved for AD without regard to severity, the use of aducanumab was quickly limited to mild AD dementia or MCI [135]. The approval of aducanumab triggered a sharp debate on the opportunity to approve a drug showing only minimal clinical benefit in a post hoc analysis of a single study [142]. The scientific community was divided by the enthusiasm for a newly introduced disease-modifying therapy and the uncommon method used to approve the drug [139]. In addition, the European Medicine Agency (EMA) refused its approval; thus, aducanumab is not approved in Europe for AD treatment [142]. The real-world safety and effectiveness of aducanumab were explored in the International Collaboration for Real-World Evidence in Alzheimer’s Disease (ICARE AD) postmarketing study, but it was terminated in 2022 due to the expected limited use of aducanumab in clinical practice, also due to medical costs [135]. Lastly, the FDA accelerated approval requires the clinical efficacy of the drug to be confirmed in trials conducted within the following nine years, until 2030. However, it is crucial to highlight the policy announced by the Centers for Medicare and Medicaid Services, which has specified that they will only provide coverage for these treatments to individuals who are enrolled in qualifying clinical trials. This decision significantly impacts the accessibility and affordability of current and future anti-amyloid antibody therapies (https://www.cms.gov/medicare-coverage-database/view/ncacal-decision-memo.aspx?proposed=N&ncaid=305; accessed on 21 May 2024).

Aducanumab’s controversial approval, its discussed clinical impact, and its subsequent decline feed the debate about anti-amyloid therapy. Aβ deposition is necessary for AD pathogenesis but seems not sufficient to cause and promote neurodegenerative changes and the corresponding cognitive decline [143]. The development of future clinical trials should not neglect the crucial interaction between amyloid, tau, and neuroinflammation, to increase the probability of clinical efficacy [143]. 

**Table 1:** Passive immunotherapy targeting Aβ plaque formation in AD. APOE: apolipoprotein E; AD: Alzheimer’s disease; ADAS: Alzheimer’s Disease Assessment Scale; DAD: Disability Assessment for Dementia; iADRS: integrated Alzheimer’s Disease Rating Scale; MCI: mild cognitive impairment; ADCOMS: Alzheimer’s Disease Composite Score; CDR-SB: Clinical Dementia Rating Sum of Boxes.

Intervening “Drug”	Trial Phase	Number of Patients Enrolled	Disease Stage	Duration	Primary Endpoint	Outcome	Authors, Year, and Country
Bapinezumab	III, placebo-controlled	1121 ε4 allele APOE carriers and 1331 noncarriers	Mild-to-moderate AD	78 weeks	ADAS-cog11; DAD	No group differences; low rate of ARIAs	Salloway et al., 2014, US [114]
Donanemab	II, placebo-controlled	257	Early symptomatic AD	72 weeks	iADRS	Mild change from baseline in the iADRS score (*p* = 0.04); high rate of ARIAs	Mintun et al., 2021, US [115]
Lecanemab	IIa, ascending dose, placebo-controlled	48	Mild-to-moderate AD	4 months	Safety and tolerability	Well-tolerated across all doses	Logovinsky et al., 2016, Sweden, US [121]
Lecanemab	IIb, placebo-controlled	854	Early AD, MCI due to AD, and mild AD dementia	12 months	ADCOMS	No group differences; low rate of ARIAs	Swanson et al., 2021, Sweden, US [122]
Ponezumab	IIa and IIb, ascending dose, placebo-controlled	77 + 26 (phase A) 63 + 32 (phase B)	Mild-to-moderate AD	18 months	Safety and tolerability	Well-tolerated across all doses	Landen et al., 2021, US [123]
Crenezumab	III, placebo-controlled	813 + 806	Prodromal-to-mild AD	100 weeks	CDR-SB	No clinical effects	Ostrowitzki et al., 2022, multicenter [126]
Gantenerumab	III, placebo-controlled	985 + 980	MCI due to AD, and mild AD dementia	116 weeks	CDR-SB	No clinical effects	Bateman et al., 2023 [117]
Solanezumab	III, placebo-controlled	2129	Mild AD dementia	76 weeks	ADAS-cog14	No clinical effects	Honig et al., 2018, multicenter [130]
Aducanumab	III, placebo-controlled	1638 + 1647	MCI and early AD	76 weeks	CDR-SB	Difference in CDR-SB in first 1638 patients	Haeberlein et al., 2022, multicenter [140]

## 4. Beyond Amyloid: Tau and Neuroinflammation 

### 4.1. Anti-Tau Therapy

Some evidence—from animal models to human brain imaging—supports the role of tau in AD pathology. From a pathological point of view, the p-tau alters microtubule stabilization and axonal trafficking, promoting the aggregates’ formation [144]. Tau deposition is associated—better than Aβ—with cognitive decline, being an interesting therapeutic target and a marker for clinical monitoring [145,146]. The first anti-tau immunotherapy was tested in 2007 in a mouse model, showing significant benefit [147], and thus translated to clinical research. Active immunization strategy is based on administering tau or p-tau protein, associated with adjuvants, for stimulating the patient’s immune response. Currently, two tau vaccines are under evaluation: AADVac1 and ACI-35. 

AADvac1 is the first tau vaccine tested in humans [148], based on an immunogen peptide triggering the production of endogenous antibodies against a 12-amino-acid sequence in the tau protein microtubule-binding region [149]. The peptide is coupled with a carrier providing T-cell epitopes without stimulating a T-cell response, thus reducing the development of severe adverse immune reactions [149]. In the phase I study, almost all mild-to-moderate AD patients developed a solid anticorpal immune response, without severe adverse events [150]. Safety has been confirmed in a long-term follow-up study, which also reported other interesting results: patients with higher levels of antibodies showed reduced hippocampal atrophy [149]—associated with the CSF level of p-tau being significantly reduced—suggesting that the vaccine affects disease pathology [151]. A more recent clinical trial confirmed the biological effect of AADvac1, but no clinical positive effects were reported [152], except for the CDR Sum of Boxes slow decline [153]. Despite the promising results, AADvac1 still needs further confirmation in larger studies with a longer follow-up.

Despite positive effects in mouse models [154], the safety and efficacy of vaccine ACI-35, targeting the pathological conformers of p-tau, are still under evaluation in mild-to-moderate AD patients.

Most anti-tau mAbs entered clinical trial research due to the significant therapeutic effects reported in animal models on both biology and clinical and functional impairment [71,155]. Using mAbs reduces the risk of uncontrolled immunological adverse effects associated with vaccines and increases specificity for anti-tau epitope response [156]. However, the disappointing results reported so far suggest that current strategies cannot obstacle tau diffusion and the spreading of neurodegenerative changes. 

Semorinemab is an anti-tau mAb binding all the six human tau isoforms. Preclinical reports showed that semorinemab reduced tau-mediated neuronal toxicity and tau deposition with a safe profile [157], but in a clinical setting in a phase II trial, it failed to improve clinical symptoms in AD patients [158,159]. In parallel, another phase II trial explored semorinemab efficacy in patients with mild-to-moderate AD, with a negative outcome [160]. Gosuranemab binds tau monomers and fibrils with high affinity, reducing N-terminal tau from interstitial fluid and CSF and lowering tau aggregation in cells [161]. When tested in NDDs, including AD and progressive supranuclear palsy [162], it failed to improve cognitive and functional outcomes [163]. Tilavonemab is an anti-tau mAb binding the tau N-terminus [164], which showed a good safety profile but no significant benefit [165]. Zagotenemab, a humanized antibody targeting extracellular aggregated tau, showed a good safety profile in a phase I trial [166], but no efficacy data are currently available. Clinical trials targeting tau are detailed in Table 2.

### 4.2. Neuroinflammation

Neuroinflammatory responses have been associated with neuronal loss and cognitive decline [167]. Microglia cells represent the first line of defense against any kind of cerebral insult and are primarily involved in neuroinflammation linked to neurodegeneration [168,169]. 

Several drugs can target the microglia, adopting different strategies [170]: (1) inhibiting activated microglia, thereby diminishing the generation of pro-inflammatory factors, chemokines, and cytotoxic substances; (2) modulating the pro- and anti-inflammatory microglia upon activation; (3) enhancing the phagocytosis mediated by microglia, aiming to eliminate detrimental tissue linked to AD; (4) targeting specific microglia subtypes associated with AD; (5) depleting and regenerating microglia to neutralize activate cells and stimulate newly formed microglia [170].

As in other neurological disorders, the first class of drugs tested in the neuroinflammatory pathway was the non-steroidal anti-inflammatory drugs (NSAIDs) [171]. NSAIDs were proposed due to the generalized anti-inflammatory effect aiming to suppress activated microglia. Epidemiological studies and meta-analyses reported conflicting results on AD development and progression [172]. Randomized clinical trials in the early 2000s tested different NSAIDs including aspirin, indomethacin, celecoxib, ibuprofen, and naproxen in AD patients, without reporting positive outcomes [173,174,175]. More recently, in 2020, a retrospective study from the Alzheimer’s disease neuroimaging initiative (ADNI) cohort, including AD, MCI, and controls, reported a decrement in AD prevalence in individuals using NSAIDs and paracetamol, thus suggesting an association independent of the anti-inflammatory effects; however, only diclofenac was found to be associated with reduced cognitive decline as measured by the MMSE score in all groups over time [176]. The crucial functions of microglia related to homeostasis foster the hypothesis that a global suppression of neuroinflammatory response might be useless in preventing NDDs.

Modulating microglia activity might be a more promising approach. Microglia express the granulocyte-macrophage colony-stimulating factor receptor (GM-CSFR). Activating the GM-CSFR generates non-inflammatory proliferation [177]. Sargramostim is a recombinant human GM-CSF stimulating bone marrow [178]. In AD, it has been shown to increase microglia proliferation and activity, supporting positive cognitive effects and plasma biomarker evidence of amyloid and tau pathology reduction [178]. The rationale on the use of sargramostim is based on the hypothesis that microglia activity should be modulated and not globally suppressed, since a bustling protective microglia population may enhance the counteraction of neurodegeneration [179].

A more sophisticated strategy considers regulating microglial phagocytosis. Aging is associated with a decline in microglial phagocytosis and deleterious microglia-mediated synaptic pruning [180,181]. The TREM2 gene is predominantly expressed in brain microglia and associated with microglial phagocytosis and synaptic pruning. Genetic variants in TREM2 are associated with a significant increase in the risk of AD [182], while TREM2 deficiency attenuates neuroinflammation and protects against neurodegeneration linked to abnormal synaptic pruning [183]. AL002 is an mAb binding to TREM2, activating microglia and Aβ phagocytosis and shown to ameliorate AD in a mouse model; in humans, AL002 has been shown to be safe and well tolerated, engaging TREM2 based on cerebrospinal fluid biomarkers [184]. DNL919 is another TREM2 antibody activating microglia and facilitating Aβ phagocytosis. No phase II trial has been scheduled due to “safety signals of moderate, reversible hematologic effects” that were “observed at the highest dose tested, suggesting a narrow therapeutic window” for AD patients [179].

Not only microglia, but other inflammatory cells participate in neuroinflammatory responses and may drive neurodegeneration. T-regs play a protective role by suppressing inflammation, but in AD, their disfunction can inhibit anti-inflammatory function, promoting a “toxic” pro-inflammatory status [185,186]. Interleukin-2 (IL-2) is a cytokine able to expand and restore functional T-regs, and interestingly, it can work as a drug. One recent phase I clinical trial involving AD patients treated with intravenous IL-2 showed a good safety profile, with no patients reporting serious adverse effects. The treatment was effective in increasing the T-reg percentage. In addition, the IL-treatment was associated with stability in cognitive and functional outcomes [187]. Another promising target is TNF-α, a cytokine which is increased in the CSF of AD patients and has been directly correlated with disease progression [188]. Although not specific to AD, the TNF-α chronic production by microglia generates a pathological inflammatory response. Interestingly, patients treated with TNF-blocking agents for other non-neurological disorders were associated with a lower risk of developing AD [189]. For cognitive decline, however, no clinical trials are ongoing. 

As already proposed for other NDDs [190], a significant innovation in this context is the development of a platform trial (NCT04795466). This new trial design permits evaluating the effect of several anti-inflammatory agents on cognition in early AD in parallel. The first molecule in the study is Canakinumab, a human anti-IL-1beta mAb that modulates neuroinflammation and inhibits the pro-inflammatory response [191]. 

## 5. Gene Therapy

Gene therapy is an innovative and promising therapy primarily focused on a genetic target but able to modify both the genetic and the molecular environment, targeting beta-amyloid plaques, reducing inflammation and the loss of brain cells, and also replacing damaged neurons. However, its role in AD is debated and controversial, with few findings able to support this approach. 

It is well known that AD is a condition that is rarely inherited (roughly 1–5% of cases). It is also true that some genetic conditions, such as Down’s syndrome, could favor the disease development [31]. It has also been demonstrated that the presence of determined alleles of apolipoprotein E (APOE) could lead to a major AD risk. APOE is a lipoprotein working as the major carrier of cholesterol within the brain [192,193]: in fact, allele ε4 of APOE is the most potent genetic risk factor for the development of AD, while APOE ε2 carriers showed a protective effect [194].

Gene transfer of APOE ε2 could be a promising treatment, but, to date, the result has not been wholly satisfying [195]. Currently, for APOE4 homozygotes patients, a phase I/II clinical trial is ongoing (NCT03634007). This study aims to evaluate the safety and toxicity of intrathecal administration of AAVrh.10hAPOE2 (LX1001), a serotype rh.10 adeno-associated virus (AAV) gene transfer vector expressing the complementary deoxyribonucleic acid (cDNA) coding for human apolipoprotein E2 (APOE2), directed to the CSF of APOE4 homozygotes patients. 

More specifically, in order to perform promising gene therapy for AD, it is important to identify specific molecules that are targetable. For example, the nerve growth factor (NGF) is a neurotrophic factor crucial for developing and maintaining hippocampal functions [195]. For its essential role in memory function, it has been proposed and tried to deliver the NGF gene with some promising results [195,196]. In 2003, Tuszynski et al., performed a phase 1 trial of ex vivo NGF gene delivery in eight mild AD patients, implanting autologous fibroblasts genetically modified to express human NGF into the forebrain. A longitudinal neurocognitive analysis suggested improvement in the rate of cognitive decline and a significant increase in cortical FDG-PET metabolism after treatment [197]. A few years later, the AAV2-NGF Study Team [198] tested if the stereotactically guided intracerebral injection of AAV2-NGF was well tolerated and exhibited preliminary evidence of impact on cognitive decline, with encouraging results regarding safety, but no positive results were obtained on clinical outcomes or selected AD biomarkers.

Another protagonist which plays a significant role in memory and learning is the brain-derived neurotrophic factor (BDNF). BDNF is a nervous system growth factor that regulates neuronal function in key memory circuits of the brain, reducing cell loss, stimulating cell function, and building new connections between brain cells in animal models. Also, experimental trials in animal models have demonstrated that delivery of the BDNF gene improved memory functions [195,199]. However, the attempts at BDNF-related therapy in animal models have shown that its injection did not correlate with spare neurons nor with levels of amyloid or amyloid plaques [195]. A phase I first-in-human clinical trial is currently ongoing (NCT05040217), to test whether a BDNF administered into the brain continuously by gene therapy can slow or prevent cell loss in the brains of people affected by MCI and AD. In this protocol, the administration of BDNF takes place through an AAV2 vector, stereotaxically administered into the brain under MRI guidance.

Another potential target was identified in neprilysin, a metallopeptidase involved in Aβ degradation [200]. In 2004, Iwata et al. demonstrated that the expression of neprilysin through injection using viral vectors could reduce Aβ levels in the region of interest [200]. Other enzymes have been studied in animal models for their ability to degrade amyloid plaques, such as cathepsin B or endothelin-converting enzyme, with hopeful results [199].

Matrix metalloprotease 9 (MMP9) is a type IV collagenase that participates in tissue remodeling by degrading extracellular matrix components. The role of MMP9 has been extensively explored due to its association with several physiological and pathological processes in the brain, where MMP9 plays multiple effects, contributing to beneficial, such as neurogenesis and axonal growth, and deleterious mechanisms [201]. In AD, high brain levels of MMP9 and CSF MMP9 levels correlated with cognitive decline have been reported [202,203]. Previous experimental studies have shown the ability of MMP9 to degrade Aβ peptides [204,205], and its regulation may represent a favorable target for stimulating Aβ clearance [206]. However, MMP9 is also involved in microglia activation, in maintaining synaptic plasticity, and in favoring non-amyloidogenic pathways [201]. In addition, MMP9 activities in AD can be influenced by the APOE genotype [207]. The complex function of MMP9 can be extrapolated from the multiple effects related to its experimental modulation in animal models: MMP9 inhibition was shown to improve specific neurobehavioral disturbances associated with AD in mice, but without affecting spatial learning and memory [208].

Lastly, promising data will hopefully be obtained from antisense oligonucleotides. BIIB080 is the first antisense oligonucleotide designed to target microtubule-associated protein tau (MAPT) mRNA and prevent the production of tau protein. In 2017, a trial with monthly intrathecal dose-escalation BIIB080 injections was started, enrolling 46 people between the ages of 50 and 74 whose mild AD was confirmed by CSF biomarkers. The trial results were recently published [209], showing a CSF total tau and p-181 tau reduction by 60 percent for six months in the higher-dose cohorts. Also, imaging results were promising, with reduced tau aggregates below baseline levels in all brain regions examined with tau PET [210]. Phase 2 is ongoing (NCT05399888). 

## 6. Stem Cell Therapy

Due to their immunomodulatory, anti-inflammatory, and regenerative properties, stem cells (SCs) can yield enduring benefits for patients with AD, as exemplified in recent studies on other NDDs, such as ALS and PD [211,212,213], serving as a springboard for further exploration and application of this approach across other disorders. Overall, there are four major SC types potentially usable for AD treatment, namely neural, mesenchymal, embryonic, and induced pluripotent SCs, but currently, only the mesenchymal stem cells (MSCs) are tested in AD patients [214]. MSCs are the most manageable cells for several reasons, including their excellent accessibility, a wide range of differentiating potential (even if they do not have the ability to differentiate into neurons), various routes of administration, and low immune response. The first clinical trial [215] with repeated intracerebroventricular injection of human umbilical cord blood MSCs (NEUROSTEM^®,^ Longeveron, Miami, FL, USA) on nine patients with mild-to-moderate AD was performed in the Republic of Korea. Except for adverse events that occurred in the first 36 h after the treatment (fever, headache, nausea, and vomiting), the transplantation was feasible, relatively and sufficiently safe, and well tolerated. Another phase I clinical trial assessing MSCs (Lomecel-B) in AD revealed encouraging results, including enhancements in cognitive function, hippocampal tropism, and fluid biomarkers, paving the way for larger phase II/III trials [215]. Despite the potential of SC therapy for AD, currently, there are still several limitations, including the lack of clinical trials with neural SCs, mainly due to the difficult related to the in vitro expansion, the risk of tumor formation, the ethical issues, the cost of this type of therapy, and the technical limitations.

## 7. Conclusions

The deposition of Aβ into pathological aggregates is considered the hallmark of AD, but it is still insufficient to generate cognitive decline and dementia. The deposition of p-tau, associated with neuronal dysfunction and death, has been more strictly correlated with the development of cognitive impairment. Neuroinflammation may play a double role, beneficial when favoring Aβ clearance and protection against insults and deleterious when chronically stimulated, feeding several pro-inflammatory pathways associated with neurodegeneration. Acting solely on misfolded protein deposition could be only a partially effective approach. However, the importance of detecting early neurodegenerative changes in a potentially reversible phase of cognitive impairment should not be neglected. The main challenges of AD therapy faced by researchers in the last two decades rely on identifying the correct targets in the optimal time window. A multimodal approach, integrating the anti-amyloid and tau therapies with the modulation of neuroinflammatory responses, might represent a successful strategy in this field. 

## Figures and Tables

**Table 2:** Passive immunotherapy targeting tau in AD. AD: Alzheimer’s disease; MCI: mild cognitive impairment; ADAS: Alzheimer’s Disease Assessment Scale; ADCS-ADL: Activities of Daily Living Inventory; CDR-SB: Clinical Dementia Rating Sum of Boxes.

Intervening “Drug”	Trial Phase	Number of Patients Enrolled	Disease Stage	Duration	Primary Endpoint	Outcome	Authors, Year, and Country
Semorinemab	Phase II	272	Prodromal-to-mild AD	48/60 weeks	ADAS-Cog11; ADCS-ADL	No clinical effects	Monteiro et al., 2023, US [160]
Gosuranemab	Phase II, dose finding	654	Early AD	78 weeks	Safety and efficacy	Safe profile and well-tolerated	Shulman et al., 2023, US [163]
Tilavonemab	Phase II	453	Early AD	96 weeks	Safety and efficacy; CDR-SB	Well-tolerated but no clinical effects	Florian et al., 2023, multicenter [165]
Zagotenemab	Phase Ib, placebo-controlled	22	MCI or early AD	49 weeks	Safety and pharmacokinetics	No adverse events	Willis et al., 2023, US [166]

## Data Availability

Not applicable.
